# Weed genomics: yielding insights into the genetics of weedy traits for crop improvement

**DOI:** 10.1007/s42994-022-00090-5

**Published:** 2023-01-09

**Authors:** Yujie Huang, Dongya Wu, Zhaofeng Huang, Xiangyu Li, Aldo Merotto, Lianyang Bai, Longjiang Fan

**Affiliations:** 1 Institute of Crop Science and Institute of Bioinformatics, Zhejiang University, Hangzhou, 310058 China; 2 Institute of Plant Protection, Chinese Academy of Agricultural Sciences, Beijing, 100193 China; 3 Department of Crop Sciences, Agricultural School Federal University of Rio Grande do Sul, Porto Alegre, 91540-000 Brazil; 4 Hunan Weed Science Key Laboratory, Hunan Academy of Agriculture Sciences, Changshang, 410125 China

**Keywords:** Weeds, Genome sequencing, Population genomics, Adaptive traits, Evolution

## Abstract

Weeds cause tremendous economic and ecological damage worldwide. The number of genomes established for weed species has sharply increased during the recent decade, with some 26 weed species having been sequenced and de novo genomes assembled. These genomes range from 270 Mb (*Barbarea vulgaris*) to almost 4.4 Gb (*Aegilops tauschii*). Importantly, chromosome-level assemblies are now available for 17 of these 26 species, and genomic investigations on weed populations have been conducted in at least 12 species. The resulting genomic data have greatly facilitated studies of weed management and biology, especially origin and evolution. Available weed genomes have indeed revealed valuable weed-derived genetic materials for crop improvement. In this review, we summarize the recent progress made in weed genomics and provide a perspective for further exploitation in this emerging field.

## Introduction

The *Arabidopsis thaliana* genome sequence was released in 2000 and represented a hallmark in plant research as the first sequenced and assembled plant genome (The Arabidopsis Genome Initiative 2000). Driven by the rapid development of sequencing technologies and bioinformatics methods, hundreds of plant genomes have since been sequenced and assembled (Sun et al. 2022a). High-quality reference genomes have provided vital resources for molecular genetics and have accelerated and improved precision crop breeding. Whole-genome genetic information for entire populations also offers accurate and plentiful molecular markers from which to infer and reconstruct the complex evolutionary histories of plant species, particularly for crop species.

Crops are not the only plants that grow in fields, however, weeds—defined here as non-crop plants growing within crop fields—can have competitive advantages over crop plants and cause yield loss (Basu et al. 2004). To date, 2847 plant species belonging to 177 families and 1118 genera have been designated as weeds (Weed Science Society of America database, http://www.wssa.net). Notably, weeds are the main contributors to yield loss for field crops, compared to pests and pathogens, and on average result in a 30% annual yield loss across the major crops (Oerke 2006). Although agricultural production is substantially affected by weeds, until recently, weed studies have not been given sufficient attention, in terms of both traditional molecular biology and genome analyses. Recent comparative genomics and population genomics analyses have revealed the effect of weeds on crop agronomic traits and the mechanisms underlying weediness, such as in barnyard grass (*Echinochloa crus-galli*), tall waterhemp (*Amaranthus tuberculatus*), and weedy rice (*Oryza sativa* f. *spontanea*) (Guo et al. 2017; Kreiner et al. 2018, 2019; Gaines et al. 2020; Qiu et al. 2020). In addition, the complex relationships among crops, weeds, humans, and abiotic environments in agricultural ecosystems, provide an ideal model for the study of biological interactions. Considering the potential of weed biology, recently, the weed research community endeavored to initiate genome sequencing of global weed species (Ravet et al. 2018).

In this review, we summarize genome sequencing of weed species over the past decade and explore future directions and potential applications in agricultural production.

## Weed genome sequencing and de novo assembly

**Fig. 1:**
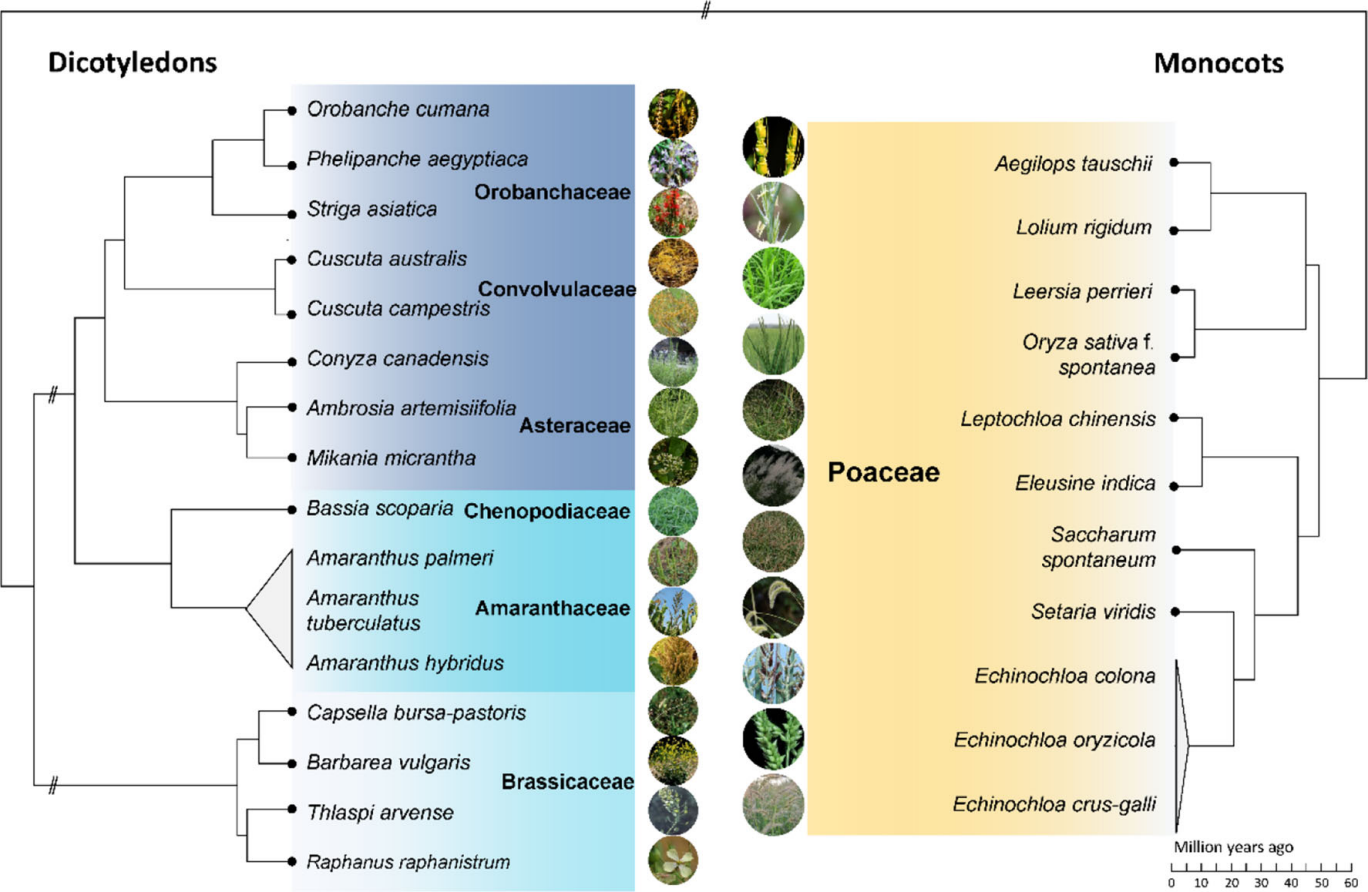
List of weed species sequenced and their phylogenetic relationships. For detailed information about all genome sequencing results, please see Table 1

In recent years, the number of genomes released for weed species has sharply increased (Table 1), with genomes for at least 26 weed species being sequenced. Their genome sizes range from 270 Mb (*Barbarea vulgaris*) to 4360 Mb (*Aegilops tauschii*); 17 of these genomes have been assembled to the chromosome level, based on long-read sequencing technologies. Meanwhile, a significant improvement in sequence quality for weed genomes was achieved along with the development of new sequencing technologies. For instance, the genomes of the barnyard grass species, *E. crus-galli* and *E. oryzicola*, which grow in paddy fields and compete with rice, were assembled into draft genomes and later anchored to chromosomes by incorporating data from chromosome conformation capture (Hi-C) (Guo et al. 2017; Ye et al. 2020; Wu et al. 2022b). The genome of weedy rice (*Oryza sativa* f*. spontanea*) was also sequenced and assembled, at the chromosome level, in 2019 (Sun et al. 2019). In addition, the genomes of tetraploid Chinese sprangletop (*Leptochloa chinensis*) were assembled (Wang et al. 2022). An invasive weed in wheat fields, field pennycress (*Thlaspi arvense*), had its genome assembled in 2015 and independently anchored to chromosomes in 2021 and 2022 (Dorn et al. 2015; Geng et al. 2021; Nunn et al. 2022). The genomes for other agronomically important weeds have also been sequenced. For example, chromosome-level genomes of highly heterozygous *Amaranthus* species (*A. tuberculatus*, *A. hybridus*, and *A. palmeri*) have been developed (Montgomery et al. 2020). Of the 26 weed species, 16 are dicots from six different families, with the remaining nine species being monocots from only one family (Poaceae) (Fig. 1). Polyploid species usually exhibit more dominant advantages in their adaptation (te Beest et al. 2012), and the genomes of four polyploid weed species, comprising three tetraploid (*L. chinensis*, *E. oryzicola*, and *Capsella bursa-pastoris*) and one hexaploid (*E. crus-galli*) species, have been sequenced. Notably, several weeds are very closely related to crop species (i.e., they represent different subspecies or accessions of the same species), and the corresponding crop genome can therefore be used as a reference genome for weeds. For example, the available barnyard millet (*E. colona* var. *frumentacea*) genome provided an important reference for barnyard grass (*E. colona* var*. colona*) (Wu et al. 2022b), as did the crop sorghum (*Sorghum bicolor*) for Johnsongrass (*Sorghum halepense*), cultivated pearl millet (*Pennisetum glaucum*) for wild pearl millet (*Pennisetum violaceum*), rye (*Secale cereale*) for weedy rye (*S. cereale* subsp. *segetale*), sugar beet (*Beta vulgaris*) for sea beet (*Beta vulgaris* ssp. *maritima*), and rice for weedy rice.

**Table 1:** Progress of de novo sequencing and assembly of weed genomes in the past decade

Year released	Common name	Scientific name	Ploidy	Genome size (Mb)	Assembly level	Contig N50 (kb)	Main crop	References
2013	Tausch's goatgrass	*Aegilops tauschii*	Diploid	4244	Scaffold	4	Wheat	Jia et al. (2013)
2014	Horseweed	*Conyza canadensis*	Diploid	326	Scaffold	21	Cotton, corn and soybean	Peng et al. (2014)
2015	Field pennycress	*Thlaspi arvense*	Diploid	343	Scaffold	20	Wheat	Dorn et al. (2015)
2017	Barnyard grass	*Echinochloa crus-galli*	Hexaploid	1340	Scaffold	1800^a^	Rice	Guo et al. (2017)
	Tausch's goatgrass	*Aegilops tauschii*	Diploid	4225	Scaffold	93	Wheat	Luo et al. (2017)
	Tausch's goatgrass	*Aegilops tauschii*	Diploid	4310	Chromosome	113	Wheat	Zhao et al. (2017)
	Shepherd’s purse	*Capsella bursa-pastoris*	Tetraploid	252	Scaffold	37	Wheat	Kasianov et al. (2017)
	Yellow rocket	*Barbarea vulgaris*	Diploid	168	Scaffold	14	Lawn	Byrne et al. (2017)
2018	Australian dodder	*Cuscuta australis*	*/*	265	Scaffold	3630	Fabaceae	Sun et al. (2018)
	Dodder	*Cuscuta campestris*	*/*	477	Scaffold	16	Fabaceae	Vogel et al. (2018)
	Wild sugarcane	*Saccharum spontaneum*	Haploid	2560	Chromosome	45	Poaceae	Zhang et al. (2018)
	*/*	*Leersia perrieri*	Diploid	267	Chromosome	50	Rice	Stein et al. (2018)
2019	Kochia	*Bassia scoparia*	Diploid	711	Scaffold	61^a^	Wheat	Patterson et al. (2019a)
	Goose grass	*Eleusine indica*	Diploid	584	Scaffold	4	Fabaceae	Zhang et al. (2019)
	Weedy rice	*Oryza sativa* f*. spontanea*	Diploid	373	Chromosome	6090	Rice	Sun et al. (2019)
	Tall waterhemp	*Amaranthus tuberculatus*	Diploid	664	Chromosome	1740	Cotton, corn and soybean	Kreiner et al. (2019)
	Witchweed	*Striga asiatica*	Diploid	472	Scaffold	16	Poaceae	Kreiner et al. (2019)
2020	Horseweed	*Conyza canadensis*	Diploid	426	Chromosome	1676	Poaceae	Lu et al. (2020)
	Bitter vine	*Mikania micrantha*	/	1350	Chromosome	1790	Cocoa, citrus and bananas	Liu et al. (2020)
	Palmer amaranth	*Amaranthus palmeri*	Diploid	408	Chromosome	2540	Cotton, corn and soybean	Montgomery et al. (2020)
	Tall waterhemp	*Amaranthus tuberculatus*	Diploid	573	Chromosome	2580	Cotton, corn and soybean	Montgomery et al. (2020)
	Smooth pigweed	*Amaranthus hybridus*	Diploid	403	Chromosome	2260	Corn and soybean	Montgomery et al. (2020)
	Green foxtail	*Setaria viridis*	Diploid	395	Chromosome	11,200	Poaceae	Mamidi et al. (2020)
	Green foxtail	*Setaria viridis*	Diploid	397	Chromosome	19,521	Poaceae	Thielen et al. (2020)
	Barnyard grass	*Echinochloa crus-galli*	Hexaploid	1340	Scaffold	1570	Rice	Ye et al. (2020)
	Barnyard grass	*Echinochloa oryzicola*	Tetraploid	946	Scaffold	1870	Rice	Ye et al. (2020)
2021	Tausch's goatgrass	*Aegilops tauschii*	Diploid	4290	Chromosome	1720	Wheat	Wang et al. (2021b)
	Tausch's goatgrass	*Aegilops tauschii*	Diploid	4075	Chromosome	2200	Wheat	Zhou et al. (2021)
	Wild radish	*Raphanus raphanistrum* ssp*. raphanistrum*	Diploid	421	Chromosome	7764	Wheat	Zhang et al. (2021)
	Wild radish	*Raphanus raphanistrum* ssp*. landra*	Diploid	418	Chromosome	4068	Wheat	Zhang et al. (2021)
	Field pennycress	*Thlaspi arvense*	Diploid	527	Chromosome	4180	Wheat	Geng et al. (2021)
2022	Field pennycress	*Thlaspi arvense*	Diploid	526	Chromosome	13,300	Wheat	Nunn et al. (2022)
	Barnyard grass	*Echinochloa crus-galli*	Hexaploid	1340	Chromosome	1570	Rice	Wu et al. (2022b)
	Barnyard grass	*Echinochloa oryzicola*	Tetraploid	946	Chromosome	1870	Rice	Wu et al. (2022b)
	Chinese sprangletop	*Leptochloa chinensis*	Tetraploid	416	Chromosome	8500	Rice	Wang et al. (2022)
	Common ragweed	*Ambrosia artemisiifolia*	Diploid	1258	Scaffold	271^a^	Tomato, lettuce and maize	Bieker et al. (2022)
	Sunflower broomrape	*Orobanche cumana*	/	1418	Chromosome	13,334	Sunflower	Xu et al. (2022)
	Egyptian broomrape	*Phelipanche aegyptiaca*	/	3877	Scaffold	9973	Cucurbitaceae	Xu et al. (2022)
	Ryegrass	*Lolium rigidum*	Diploid	2440	Chromosome	361,790^a^	Wheat	Paril et al. (2022)

^a^Scaffold N50 size (kb)

*/*Data missing

## Whole-genome sequencing of weed populations

Whole-genome sequencing, which provides excellent tools for mining genetic mechanisms and evolutionary studies, has been widely used in crop genomics (Jia et al. 2021). Since 2017, this method has also been applied to a limited number of weed species, mainly for paddy weeds, such as weedy rice and barnyard grass (Table 2).

**Table 2:** Summary of recent investigations on weed populations by genome resequencing

Common name	Scientific name	Population size	Sequencing depth	Region	References
Weedy rice	*O. sativa* f*. spontanea*	38	19 ×	USA	Li et al. (2017)
		155	18 ×	China	Qiu et al. (2017)
		30	10 ×	Korea	He et al. (2017)
		331	19 ×	Global	Qiu et al. (2020)
		50	23 ×	Japan	Imaizumi et al. (2021)
		48	40 ×	USA	Wedger et al. (2022)
Barnyard grass	*E. crus-galli*	328	15 ×	China	Ye et al. (2019)
		578	15 ×	Global	Wu et al. (2022b)
Barnyard grass	*E. walteri*	15	15 ×	USA	Wu et al. (2022b)
Barnyard grass	*E. colona* var*. colona*	20	15 ×	Global	Wu et al. (2022b)
Barnyard grass	*E. oryzicola*	85	15 ×	Global	Wu et al. (2022b)
Tall waterhemp	*A. tuberculates*	173	10 ×	USA	Kreiner et al. (2019)
Green foxtail	*S. viridis*	598	43 ×	Global	Mamidi et al. (2020)
Fonio millet	*D. longiflora*	17	20 ×	Africa	Abrouk et al. (2020)
Field pennycress	*T. arvense*	40	19 ×	China	Geng et al. (2021)
		40	15 ×	USA	Nunn et al. (2022)
Chinese sprangletop	*L. chinensis*	89	19 ×	China	Wang et al. (2022)
Weedy rye	*S. cereale* subsp*. Segetale*	30	10 ×	Global	Sun et al. (2022a)
Common ragweed	*A. artemisiifolia*	655	24 ×	USA	Bieker et al. (2022)

Weedy rice was the first weed species to be used for genomic investigation, via whole-population sequencing. As weedy rice can be considered a wild-like rice ecotype, the genome of cultivated rice provides a good reference for calling single-nucleotide polymorphisms (SNPs) in individuals. Over 650 accessions of weedy rice have been sequenced, being derived from global rice production areas, which has deepened our understanding of weedy rice origins and adaptation strategies (Li et al. 2017; Qiu et al. 2017, 2020; Imaizumi et al. 2021; Wedger et al. 2022).

Other weeds affecting paddy fields have also been studied, at the genomic level. For barnyard grass, the release of its genome (Guo et al. 2017) heralded the beginning of population genomics in this species, with over 700 genomes of accessions collected from all over the world being re-sequenced for studies on evolutionary history and typical weed adaptation syndromes (Ye et al. 2019, 2020; Wu et al. 2022b). Similarly, genome resequencing of 89 Chinese accessions revealed that sprangletop originated from a local population in tropical areas of South Asia and Southeast Asia and that the geographical range of individuals with herbicide resistance genes expanded, likely due to field management practices (Wang et al. 2022).

In recent years, significant efforts have been made to explore the adaptation and evolutionary dynamics of field pennycress. For example, 40 field pennycress lines from different altitude regions were re-sequenced, resulting in the identification of one SNP responsible for the adaptation to latitude, via constructing ultra-high-density linkage maps (Geng et al. 2021). In another example, a genomic region located on scaffold 6 was identified as causing the seedling color phenotype in field pennycress by bulk-sequencing of DNA pools from 20 wild-type and 20 pale plants (Nunn et al. 2022).

## Genomic insights into weed biology

### Environmental adaptation

Weeds have great potential as model systems in which to understand plant responses to biotic and abiotic stresses (Vigueira et al. 2013). They can survive in disrupted environments and persist under multiple challenges, in particular escaping from control measures in the field, including targeted tillage practices, herbicide use, and hand-weeding (Sharma et al. 2021; Neve and Caicedo 2022). In addition, weeds are not distributed in limited ecological niches, but rather, they often exhibit a widespread distribution, even among areas with distinct conditions, exemplifying their strong environmental plasticity (Sharma et al. 2021).

Genomic studies have significantly improved our understanding of weed environmental adaptations to biotic and abiotic stresses. For example, *T. arvense* is an annual weed from the Brassicaceae family that lives at different altitudes, ranging from sea level to 4500 m above sea level. Genomic analyses of populations from different ecological conditions identified a SNP that led to a loss-of-function allele in *FLOWERING LOCUS C* on chromosome 1, which contributed to the early flowering trait that was key to the success of high-elevation populations (Geng et al. 2021).

Another conspicuous trait related to environmental adaptation in weeds is herbicide resistance (Hawkins et al. 2019; Gaines et al. 2020). Comparative genomics between herbicide-susceptible and -resistant individuals, from the same species, and between species, can offer glimpses into innovations in herbicide resistance pathways (Kreiner et al. 2018). Waterhemp (*A. tuberculatus*), which is troublesome in maize (*Zea mays*) and soybean (*Glycine max*) fields, is notorious for exhibiting multiple herbicide-resistant (MHR) traits. Recently, a reduction–dehydration–glutathione (GSH) conjugation system was discovered as a possible pathway for MHR (Concepcion et al. 2021). In palmer amaranth (*Amaranthus palmeri*), genomic analysis helped determine that herbicide resistance is conferred by an extrachromosomal circular DNA (eccDNA) of about 400 kb in length that harbors *5-ENOYLPYRUVYLSHIKIMATE-3-PHOSPHATE SYNTHASE* (*EPSPS*), which encodes the enzyme targeted by the herbicide glyphosate (Gaines et al. 2010; Molin et al. 2020). Although the amplification of genes and gene clusters, via eccDNAs or other structures, is a common stress-avoidance mechanism in plants (Nandula et al. 2014; Singh et al. 2020), it is usually transient and not stably inherited (Lanciano et al. 2017; Gaines et al. 2019).

As the most dominant weed in rice fields, barnyard grass has also evolved global resistance to major herbicides. Genome resequencing of barnyard grass individuals from Brazil, Italy, and China revealed four mutations in the gene encoding aceto-lactate synthase (ALS), which conferred herbicide resistance, namely Ala-122-Thr, Trp-574-Leu, Ser-653-Asn, and a Gly-654-Cys substitution identified for the first time, with a tendency to occur in sub-genome A (barnyard grass is a hexaploid). Moreover, after comparing the genomes of resistant and susceptible individuals from Brazil, an Arg-86-Gln mutation in the conserved degron tail region of *Echinochloa* AUXIN-INDUCED (AUX)/INDOLE-3-ACETIC ACID INDUCIBLE 12 (IAA12) was identified, which has since been confirmed to confer resistance to other auxin-like herbicides (LeClere et al. 2018; Figueiredo et al. 2021; Wu et al. 2022b).

**Fig. 2:**
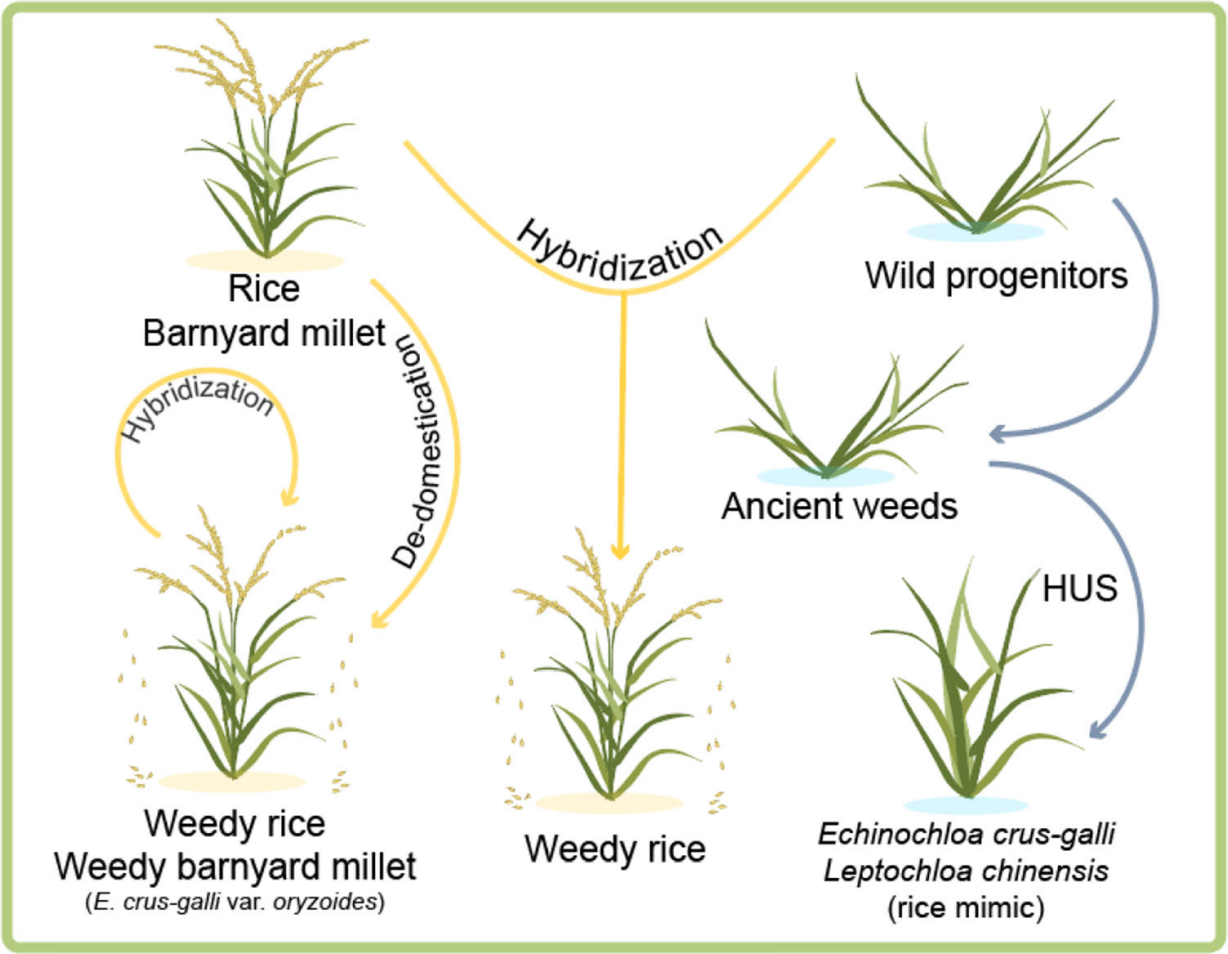
Possible origination routes for three notorious paddy weeds in rice fields, as supported by recent genomic studies. Wild progenitors include wild *Oryza*, *Echinochloa*, and *Leptochloa* species in the grass family. HUS, human unintentional selection

Great progress has also been made in understanding the responses of weeds to biotic stresses. Before herbicides were used in agriculture, the direct interaction between weeds and human beings was through hand-weeding, which placed high pressure on weed morphology, especially plant architecture. One example is the Vavilovian mimicry or crop mimicry seen in barnyard grass (at least in *E. crus-galli* and *E. oryzicola*), an unintentional human selection (UHS) resulting from human action (Fig. 2).

Crop mimicry describes the adaptation of a weed through its acquiring some of the morphological characteristics of neighboring domesticated crops, at a specific stage of their life history, to escape their removal by hand-weeding (Barrett 1983; Ye et al. 2019). The preadapted plants, or wild species that were first to colonize in cultivated fields, during the early agricultural stage (so-called ancient weeds), gradually became mimic weeds under strong artificial (weeding) selection. Genomic signatures of human selection on crop mimicry were elucidated by comparing the genomes of mimetic and non-mimicry lines of barnyard grass collected from paddy fields in the Yangtz River basin, China (Ye et al. 2019). Several genes underlying plant architecture (e.g., tiller angle) were identified, including *LAZY1*, a gene responsible for plant tiller angles, which was also under selection during rice domestication. The genomic study of mimicry of rice seedlings, by barnyard grass, is an example of how weeds can adapt to disturbed environments with selective pressure from human beings, via a genomic approach.

Allelopathic secondary metabolites also are a representative response of weeds to biotic stress. Benzoxazinoids, which acted against microbial pathogens and neighboring plants, were identified in a multitude of species of the family Poaceae, such as maize, wheat (*Triticum aestivum*), and barnyardgrass (Frey et al. 2009; Wu et al. 2022a). As a predominant representative of benzoxazinoids in plants, DIMBOA is present in barnyard grass with multiple copies and inhibits plant height and fresh weight of neighboring rice (Guo et al. 2017). Another example is momilactone A, which has similar functions to Benzoxazinoids in rice. Based on the momilactone A biosynthesis genes of rice, a syntenic gene cluster was identified in barnyard grass. Up-regulated expression of *MAS* and *KSL4*, within this cluster, under fungal infection indicated its contribution to resistance to blast infection in the paddy environment (Guo et al. 2017).

### Origins of weeds

Understanding the origin of agricultural weeds is crucial to their proper management. Weed origins can be via several routes. Preadapted plants or wild species can colonize cultivated fields in human-made ecological niches (Larson et al. 2014). With the expansion of cultivated fields, the emergence and diversification of weeds may have resulted from hybridization between crop and wild groups, along with other routes (Iriondo et al. 2018; Janzen et al. 2019).

Recent genomic studies focused on paddy weeds revealed many interesting insights about their possible origin(s) and evolution (Fig. 2). Weedy rice (*Oryza sativa* f*. spontanea*) has attracted much attention for its origin of de-domestication, i.e., the conversion of a domesticated form to a wild-like form (Wu et al. 2021). Weedy rice mimics rice cultivars, at the seedling stage, while retaining wild phenotypes, such as strong seed dormancy and shattering. De-domestication from cultivated rice (including cultivars and landraces) is the main route for rice feralization, along with introgressions from wild rice, which is commonly seen in Southeast Asia and South China, where wild rice is distributed, as well as inter-subspecies hybridization (Stewart 2017; Sun et al. 2019; Qiu et al. 2020; Wu et al. 2021). Genomic mining, aided by comparisons between the genomes of weedy, wild, and cultivated rice populations, has revealed distinct differentiation regions on chromosomes during de-domestication compared to those resulting from domestication, with the identification of a genomic island possibly underlying feralization traits on chromosome 7. This genomic region harbors *Rc*, controlling red pericarp and seed dormancy (Sweeney et al. 2006), and several tandem-duplicated genes encoding seed storage proteins (Li et al. 2017; Qiu et al. 2020).

A similar process was also described for the origins of *E. crus-galli* var*. oryzoides*, which is currently regarded as a paddy weed (Fig. 2). The significantly lower nucleotide diversity, longer linkage disequilibrium decay, more immune response genes, larger grains, and non-shattering spikelets in this species, compared to weed populations, indicate that var*. oryzoides* is an abandoned crop (Wu et al. 2022b).

## Perspectives in weed genomics

We need complete, contiguous, and accurate genome assemblies for many more weed species. Indeed, in notable contrast to the massive increase in sequenced crop genomes, only 26 weeds have been decoded thanks to the sequencing and assembly of their genomes. The enormous gap between crops and weeds underscores how much weeds are currently being overlooked. For example, Commelinales, with about 750 extant species, including pickerel weed (*Monochoria vaginalis*), are important weeds growing in paddy fields. Likewise, common water hyacinth (*Eichhornia crassip*) is the most common invasive plant according to a survey by the Weed Science Society of America database (WSSA, http://www.wssa.net). Yet, these two species still lack a representative genome. Several sedges (e.g., *Cyperus*, *Scirpus*, and *Fimbristilis*) are found worldwide and exhibit particular weediness traits, but very little genomic information is currently available.

We even lack a thorough understanding and characterization of notorious weeds affecting croplands, such as hairy crabgrass (*Digitaria sanguinalis*), a typical upland weed growing in maize and soybean fields. Moreover, a higher-quality genome of weeds is required to shed light on related biological topics. The gap-free genomes of many plants, such as *Arabidopsis*, rice, and watermelon (*Citrullus lanatus*), have recently been assembled, providing the first complete genome structure of any plant (Song et al. 2021; Wang et al. 2021a; Deng et al. 2022). With the incorporation of sequences from highly repetitive regions and centromeres into genome assemblies at the chromosome scale, a greater understanding of the global pattern of weed polymorphisms and the genetic basis of their weedy traits and high adaptability is finally within reach, but only if more genomes are sequenced or improved upon. These issues were also noted by the International Weed Science Consortium, which has designated Plantae (www.plantae.org) as a platform for community collaboration efforts and has developed a weed genomics website (www.weedgenomics.org) (Ravet et al. 2018).

We expect and anticipate more studies exploring the population genomics of weeds, which will be helpful for the understanding of their evolutionary strategies and evolutionary ecology, while offering more options for weed management. Current evolutionary patterns tend to highlight pressure imposed by the natural environment, perhaps neglecting the role that human activities play in a novel ecosystem labeled with specific species assemblages and environmental factors. Studying weed populations with complex evolutionary trajectories of traits will enhance our ability to decode their distinct evolutionary strategies under different conditions. In addition, a better understanding of the evolution of agricultural weeds will be crucial to weed management. Given the increasing number of rapid weed adaptations, such as herbicide resistance, ongoing selection for other weedy traits should be a driving force to adjust all weed management practices to mitigate the spread and success of weeds.

With the advantage of more available genomes, weed functional genomics will step to the front stage. Our understanding of the mechanisms by which multiple weed species acquire herbicide resistance (particularly non-target resistance) to the same class of herbicide has considerably improved with released genomic information (Devine and Shukla 2000; Yuan et al. 2007; Délye 2013; Kreiner et al. 2018). For example, the availability of the barnyard grass genome made it possible to identify, for the first time, a significant increase in copy number for cytochrome P450 genes in the weed genomes, as well as a Gly-654-Cys substitution, with both strategies contributing to ALS resistance. Another example resulting from the comparative analysis of waterhemp genomes was the report of a possible pathway for MHR, via reduction–dehydration–glutathione. We anticipate that, along with the development of weed genomics, additional discoveries about gene functions and their interactions will be forthcoming.

More valuable genetic resource of weeds will be revealed with the sequencing of more weed genomes, which will have benefits for the genetic improvement of crops and even their de novo domestication. Crops, particular orphan crops, are genetically very closely related to weeds. For example, orphan crops usually have a notorious weed species in the same genus (Ye and Fan 2021). Given their strong environmental plasticity and high level of genetic variation, weeds are an untapped genetic resource for domestication. For example, mutating the orthologs for *qSH1* (*Shattering QTL 1*) and *Sh4* (*Shattering 4*) genes in weeping rice grass (*Microlaena stipoides*), an Australian wild relative of rice, caused the loss of shattering in this species (Shapter et al. 2013). Historically, some weeds have been domesticated into crops, such as rye (*Rye secale*) (Sun et al. 2022b). Presently, de novo domestication of new crops is an option being considered to mitigate the effects of climate change on global crop production. We propose that some weeds, in particular those mimicking crops, are ideal targets for de novo domestication.

In addition to crop improvement, weed management will also benefit from the advances in weed genomes. Gene silencing techniques are offer a promising approach to manipulate the expression level of weed traits genes to reduce their impact with improved understanding of characteristic regulated pathways (Neve 2018). For example, if genomics can identify the basis of allelopathy, weeds could be modified with low levels of allelopathic compounds, thereby reducing their competitive ability in paddy fields. However, major challenges remain to be overcome; e.g., the designation of highly specific gene silencing triggers with high heritability (Patterson et al. 2019b).

Post-transcriptional silencing, using exogenous application of RNA, known as spray-induced gene silencing (SIGS), is a promising technology that may revolutionize weed control. Several limitations and opportunities are associated with the development of this technology. The main requirement for SIGS is selective gene silencing in weeds and the absence of effects on crops and non-target organisms. Therefore, the development of this non-transgenic, and environmentally safe, technology depends largely on genome sequencing, chromosome-level assemblies, and deep knowledge of gene function for all weed species, which affect food production, and the crops whose fields they invade.

## Data Availability

The datasets generated during and/or analyzed during the current study are available from the corresponding author on reasonable request.
